# Interactionism, Post-interactionism, and Causal Complexity: Lessons From the Philosophy of Causation

**DOI:** 10.3389/fpsyg.2021.590533

**Published:** 2021-03-30

**Authors:** María Ferreira Ruiz, Jon Umerez

**Affiliations:** ^1^Department of Philosophy, University of Bielefeld, Bielefeld, Germany; ^2^Department of Philosophy, University of the Basque Country (UPV/EHU), Donostia/San Sebastián, Spain

**Keywords:** interactionism, causal parity, causal selection, nature-nurture, inter-identities

## Abstract

In biology and philosophy of biology, discussing the notion of interaction leads to an examination of *interactionism*, which is, broadly speaking, the view that rejects gene-centrism and gene determinism and instead emphasizes the fact that traits of organisms are always the result of genes and environments. It has long been asserted that the nature-nurture problem requires an interactionist solution of sorts, the so-called *interactionist consensus*. This consensus, however, has been deemed insufficient and challenged by several authors triggering an extension of the debate among contestants and defenders. Unfortunately, part of the problem is that the views on causation that would ground claims about interactionism are not always made explicit in this debate, which renders those views somewhat complicated to assess. Moreover, it seems to be assumed that causal complexity *excludes* the possibility of characterizing, distinguishing, or comparing among causal contributions. By turning to a detailed survey of the origin of the debate and to some developments in the philosophy of causation, we will contend that this view is unwarranted, and that much of the debate around interactionism is based on the drawing of this (wrong) conclusion. We also examine implications of this analysis for the project to develop a framework based on the notion of inter-identities.

## Introduction and Motivation: Biological Inter-identities

This paper responds to the challenge posed by the *Research Topic “Inter-identities’ in Life, Mind, and Society”* which the call for papers (CfP, 2019) identifies as the “struggle to understand and model complexity in the living, the cognitive and the social domains” since the systems exhibiting such complexity are thought not to be easily amenable to “classical analytic and reductionist approaches.” The alternative we are urged to explore in this special issue amounts to being able to account for those systems in terms of their “interaction with other systems and the environment.” We attempt here to simultaneously reveal and warn against the potential and latent unawareness about the issues, difficulties and debates that haunt such an alternative.

In biology and philosophy of biology, discussing the notion of interaction leads to an examination of *interactionism*, which is, broadly speaking, the view that rejects gene-centrism and gene determinism and emphasizes the fact that traits of organisms are always the result of genes and environments. It has long been asserted that the nature-nurture problem requires an interactionist solution of sorts.

There is an ongoing debate around interactionism with a somewhat dizzying dialectics. First, it is not very clear who the opponent of interactionism really is. This should be evident from the moment that many refer to interactionism as consensus view, “the interactionist consensus.” Moreover, whilst there are some who share the spirit of interactionism, they hold the belief that it falls short of overcoming fundamental issues and an alternative perspective is required. These often regard themselves as *critics* of interactionism and adopt different names (here, we will call this the “post-interactionist” trend). There are also objections against those claims that interactionism is “enough,” that is, criticisms against criticisms of interactionism that seek to restore the interactionist stance.

There are important meta-problems surrounding the nature-nurture debate. There are complaints that there seems to be, as a matter of fact, an interactionist consensus that almost nobody challenges but which seems to be still poorly understood. Evelyn F. Keller neatly identifies a problem in the debate (Keller, 2010, p. 1). She has pointed out that one of the most remarkable features of the nature-nurture debate is how often it drives us into two apparently contradictory outcomes. One is the repeated announcement that the debate has been solved, precisely, through the general acquiescence that the answer simultaneously requires both aspects, whilst the other is the persistence of the discussion, nonetheless.

Other authors have also confronted previously this paradoxical situation of the (dis)solution of a problem that comes back continually and have been forced to make an effort of clarification in order to show that, though they propose to overcome the debate along these lines, the mere appeal to interaction, just a plain “both are necessary,” without any further development or precision, does not simply leave the problem unresolved but rather contributes to its perpetuation.

In this paper, we analyze various views and show that a main concern across such views is causal, even when adequate causal analysis is not usually invoked in the literature on the topic. Indeed, the core of the problem around interactionism is how to deal with causal complexity. Causal complexity is characterized by what is sometimes called polygeny (Molnar, 2003), that is, the fact that effects are typically brought about by multiple causes, and interaction, that is, the fact that causal factors do rarely, if ever, contribute with independence from other factors. Interactionism in general accepts this, but fails to move beyond simple statements of this very kind. Unfortunately, part of the problem is that the views on causation on which claims about interactionism would be grounded are not made explicit in this debate, which renders those views somewhat complicated to assess. Moreover, it seems to be assumed that causal complexity *excludes* the possibility of characterizing, distinguishing, or comparing among causal contributions. By turning to developments in the philosophy of causation, we will contend that this view is unwarranted, and that much of the debate around interactionism is based on the drawing of this (wrong) conclusion.

Finally, we intend to examine to what extent the difficulty in elaborating a substantive notion of interaction (interactionism), beyond the mere appeal to the necessity of taking into account diverse factors when trying to understand the organism, has any implications for the project to develop the idea of inter-identity in these and other fields. Our claim is that it does have such an impact and that accordingly we cannot ignore the exigency to cope with this in explicit terms in our theories and explanations.

The paper is structured as follows. Section “The interactionist consensus” outlines the so-called interactionist consensus, an alleged default view in biology and philosophy of biology. Section “Objections to Interactionism and Post-interactionist Alternatives” summarizes the main objections to standard interactionism and outlines two alternative proposals: Susan Oyama’s *constructivist interactionism* and Richard Lewontin’s *dialectical biology*. Next, in section “Vindications of Interactionism,” we address vindications of the standard interactionist stance put forward by Philip Kitcher and Kenneth Schaffner. The consideration of these views leads us into debates on the philosophy of causation surveyed in section “Discussion: Bringing the Philosophy of Causation to the Debate,” where we make our case that the interactionism debate in philosophy of biology tends to draw the wrong pessimistic conclusion from the recognition of causal complexity. In order to understand why and search for alternatives, first sub-section “Clarifying the Original Confusion at Source of the Debate” draws on Keller historical survey on the origin and transformation of the debate and, then, sub-section “The Problem of Causal Selection” frames her moral in the general issue of causal selection, and finally sub-section “Interactionism, Post-Interactionism, and Causation” briefly introduces, respectively, epistemic and ontological options to consider. Lastly, in section “Conclusion and Prospects for the Project,” on the basis of our analysis, we draw some conclusions and we discuss the implications of this work for the *Inter-Identities* project.

## The Interactionist Consensus

The interactionist consensus emerges in opposition to what is perceived as tradition, characterized by an overarching gene-centrism. In such gene-centric tradition, biological phenomena pertaining both to evolution and to development can be accommodated in a series of dichotomous categories:

**Table:** 

Biological	Cultural
Nature	Nurture
Inherited, innate traits	Acquired, learned traits
Internal causes	External causes
Genes	Environment

Where the gene/environment distinction in the table above assumes a few other forms:

**Table:** 

Genes	Environment
Information carriers	Material support
Replicators	Interactors
Controllers	Controlled
Instructive	Permissive
…	…

Disease etiology provides a good example of the dichotomy in action. Whilst some diseases are said to be genetically determined, or innate (the terminology varying with context), others are thought to be acquired, not determined genetically (e.g., Huntington’s disease is classified as genetic, whereas type 1 diabetes is considered to be non-genetic). In a contemporary setting, the dichotomic disease (etiology) classification does not resist much pressure. Virtually any researcher would acknowledge that genes “alone” cannot determine, cause, produce, or generate anything, and that they never act alone. Interactionism opposes genetic determinism of traits and a gene-centric tradition in biology. In this sense, genetic determinism is currently not a popular stance, and anyone would instead take pride in being an interactionist. Interactionism is the position that highlights the fact that traits of organisms are always the result of genes and environments. While interactionism is “boosted” by new empirical findings in cutting-edge fields, such as epigenetics, it nevertheless emerged before and independently of such recent achievements (Kronfeldner, 2009).

Notably, finding explicit and well elaborated characterizations of interactionist views (let alone of a philosophical style) is not an easy task^1^. This is perhaps precisely because it constitutes “a consensus” (Schaffer, 2016), or a sort of *default* position in contemporary research. It is clear, however, that a certain smooth continuity holds between non-interactionist views (e.g., genetic determinism) and interactionism: the latter preserves a strong dichotomous thinking (cf. Sterelny and Griffiths, 1999; Kronfeldner, 2009). Even if the innate/acquired dichotomy no longer seems to apply, as any trait is conceived as the result of different kinds of factors, it is precisely these kinds of factors that become a prominent and irreducible dichotomy. Indeed, the very formulation of an interactionist stance rests upon the partitioning of biologically relevant factors into genetic and non-genetic. This constitutes an important line of objections to interactionism by several authors who identify the dichotomous thinking as the ultimate problem underlying much of contemporary research and theorizing in biology. In the next section, we turn to such criticisms.

## Objections to Interactionism and Post-Interactionist Alternatives

In this section, we will outline the main objections raised against traditional interactionism and the alternative (i.e., post-interactionist) views they have motivated. We will consider two such post-interactionist approaches: the so-called *constructivist interactionism* and the *dialectical biology* view.

### Interactionism as a Perpetuation of Fundamental Dichotomies and Their Asymmetry

As we advanced in the previous section, the interactionist solution is regarded with suspicion. Post-interactionist approaches target both traditional gene-centric evolutionary and developmental biology, but also the more recent “interactionist consensus” (cf. Sterelny and Griffiths, 1999; Kronfeldner, 2009) that, in their view, falls short of correcting the former’s bad habits. In that traditional view, which extends to contemporary biology, biological form is to be explained, in a kind of regressive manner, by pointing to a previous instance of form. Critics denounce that, unfortunately, this kind of explanation is not amended in regular interactionist replacements. This indictment is evidenced in this very famous warning^2^ by Oyama:

*“But wait,” the exasperated reader cries, “everyone nowadays knows that development is a matter of interaction. You’re beating a dead horse.” I reply, “I would like nothing better than to stop beating him, but every time I think I am free of him he kicks me and does rude things to the intellectual and political environment. He seems to be a phantom horse with a thousand incarnations, and he gets more subtle each time around. What we need here, to switch metaphors in midstream, is the stake-in-the-heart move, and the heart is the notion that some influences are more equal than others, that form, or its modern agent, information, exists before the interactions in which it appears and must be transmitted to the organism either through the genes or by the environment. This supports and requires just the conceptions of dual developmental processes that make up the nature-nurture complex. Compromises don’t help because they don’t alter this basic assumption”* (Oyama, 2000), p. 26–27).

The point is that, in principle, interactionism considers such a dichotomous view to be a mistaken one because, as we have seen in section “The Interactionist Consensus,” it splits the empirical world into unwarranted (if not arbitrary) realms but, also, because it privileges one of the sides at the expense of the other. What the post-interactionist authors to be introduced next criticize is that, if interactionism arose as a corrective to the faults of dichotomous views, its standard versions do not actually achieve its goal. Their indictment is that interactionism leaves those categories untouched specifically by not addressing both the issue of how the allegedly interacting poles are defined and, mainly, the remaining problem of attributing a privileged status as essential cause to one of them. In their view this amounts to perpetuating the dichotomous approach that interactionism was supposed to supersede (Griffiths and Gray, 1994, p. 277).

Therefore, we have seen that interactionism has been widely accepted as a response to traditional dichotomous views, but objections extend to interactionism as well. According, for instance, to Developmental Systems Theory (DST) supporters, biologists and philosophers with “good manners” would call themselves *interactionist*s and stop looking for either the genetic or the environmental origin of a trait. Rather, interactionists would stress the mutual influence of genetic and environmental factors and the more general nature/nurture debate would resolve in a *quantitative* way: “The question is no longer whether intelligence is innate or acquired, but instead whether intelligence is 50 percent or 70 percent genetic. DST rejects the attempt to partition causal responsibility for the formation of organisms into additive components. Such maneuvers do not resolve the nature/nurture debate; they continue it” (Oyama et al., 2001, p. 1).

In this way, judged from a critical stance, interactionism reinforces the mistake while seeming to correct it. In claiming that all phenotypes are the joint product of genes and environment, it retains all the opposing categories of the tradition and partitions causal responsibility in a very biased way: “two classes of developmental resources: genes and the rest” (Griffiths and Gray, 1994, p. 277). Things interact, interactions are important, but interactions necessitate distinct things that interact. As an example of such a conservative way of thinking, consider views that place information in loci other than genes, e.g., in the environment. Information, whether in the genes or in environments, still pre-exists, and it still presupposes two kinds of information sources, genetic, and environmental. Thus, DST places interactionism within the more traditional views.

### Oyama’s *Constructivist Interactionism*

In contrast, Oyama is much more radical. The solution for her is not to stress how nature interacts with nurture, how genes interact with the environment or where information is located. Oyama (and DST) calls the very distinctions above into question and her view is therefore an attempt to do biology without such oppositions. She seeks for a way of thinking about development that does not rely on a dichotomous perspective, even implicitly, because she does not accept pre-established and extant inter-acting instances. This is why, lately, she and other proponents of DST have assumed the label of “constructivist interactionism” in order to differentiate themselves from a more standard and flawed kind of interactionism.

In Oyama’s view this is centrally an issue about genetic determinism. She thinks that genetic determinism is already inherent in the way we (commonly) understand what genes are and how they work, i.e., an understanding of them as containing a pre-existing information. Thus, for Oyama, the solution to this contemporary preformationism does not lie in opposing some contemporary epigenetic approach. Neither preformationism nor epigenetics were satisfactory in the past and will not be today. In her view, then, we need a novel view of ontogenesis, in which information is not pre-contained somewhere outside or inside the organism ready to be used, but where information itself develops, has an ontogeny, a developmental history. Information neither pre-exists nor arises from disorder and chaos. This is why DS theorists also claim that the nature/nurture debate is not dead and explicitly reject the option to distribute causes and add them up, as we have seen before (Oyama et al., 2001, p. 2),

Oyama and other DS theorists have advocated for the so-called causal parity thesis, which has been formulated in various ways. For instance, Oyama writes that she argues for “a view of causality that gives formative weight to all necessary influences, since none alone is sufficient for the phenomenon or for any of its properties.” (Oyama, 2000), p. 15). In turn,

*“There is a fundamental symmetry between the role of the genes and that of the maternal cytoplasm, or of childhood exposure to language. The full range of developmental resources represents a complex system that is replicated in development. There is much to be said about the different roles of particular resources. But there is nothing that divides the resources into two fundamental kinds. The role of the genes is no more unique than the role of many other factors”* (Griffiths and Gray, 1994, p. 277).

We will return to causal parity in section “Vindications of Interactionism,” and particularly in sub-section “The Problem of Causal Selection,” when we address interactionism from the causal point of view. But first, we need to consider a related approach.

### Lewontin’s *Dialectical Biology*

A related but distinctive approach is that of Lewontin and Levins (and collaborators). Through the years Lewontin (1982, 1983) has developed a perspective founded on the mutual “construction” of both the organism and the environment, instead of a lineal vision consisting on the adaptation of an organism to a niche that is taken as given, and has later rounded out it with the inclusion of the gene within a triadic approach (Lewontin, 2000a: *The Triple Helix. Gene, Organism and Environment).* Consequently, in this last survey, both the relation gene-organism and the relation organism-environment are equally questioned. Moreover, the reexamination made by Lewontin of the three items included in the title of his book puts them explicitly in connection with the necessity to also develop an appropriate dialectics of “parts and wholes” or, in other terms, with the necessity to relate levels of organization and articulate what inter-level relations are and what do they imply (Umerez, 1994, 2016).

A dynamic understanding of this framework of relations amounts to the approach that Levins and Lewontin (1985) identified as *dialectical biology* (1985), an approach that implies, at least, the following tenets. First, the reconsideration of the way to understand causes, which invites us to go beyond the analysis of causes through linear models and reminds us that in genetics and development we cannot truly partition the amount of variation in a phenotype into different causes or treat several causes as additive in the production of an effect. In this sense, we cannot treat causes as independent and separable from each other. Lewontin already said in his landmark article on *the analysis of variance and the analysis of causes* that “[I]f an event results from the joint operation of a number of causative chains, and if these causes “interact” in any generally accepted meaning of the word, it becomes conceptually impossible to assign quantitative values to the causes of that individual event. Only if the causes are utterly independent could we do so” (Lewontin, 1974, p. 402). Unfortunately, the latter is not the case in the biological realm.

Secondly, they warn us about certain assumptions that also pervade interactionist perspectives and that tend to separate the organism from the environment, not considering the effect of the former on the latter, and to allocate ontological priority to the individual over the collective (in human sociality) (Lewontin et al., 1984, p. 270). A third aspect central to this view is the necessity to incorporate explicitly an analysis of levels of organization and of the relations among those levels (Lewontin et al., 1984, p. 277ff). Finally, a fourth aspect urges us to take into account the impact of random effects on development (developmental noise) (Lewontin, 2000a, pp. 17–18, 38).

Lewontin, in his “Foreword” to the new edition of Oyama (2000), characterizes the “usual interactionist view” as asserting that “there are separable genetic and environmental causes, but the effects of these causes acting in combination are unique to the particular combination,” a characterization he finds unsatisfactory since it does not challenge “the ontologically independent status of the causes as causes, aside from their interaction in the effects produced” (Lewontin, 2000a, p. xiv). This is why Lewontin, even if he thinks that Oyama’s analysis of *the ontogeny of information* does indeed overcome such a shortcoming, prefers to maintain a more clear departure: “It is this claim about causes that Oyama, in this new edition, calls “constructivist interactionism,” but that I would characterize as *dialectical* in order to emphasize its radical departure from conventional notions of interaction” (Lewontin, 2000b, p. xv).

## Vindications of Interactionism

In the previous section, we have summarized two representative, and related, sorts of criticism to interactionism and we will now present two equally representative answers to those criticisms implying some kind of qualified defense of interactionist claims. They are, moreover, two lines of response that enter in direct and explicit discussion with those very criticisms.

### The Heuristic Value of Post-interactionism

Philip Kitcher is a well-known critic of DST and related views. He defends an idea of causal democracy while also vindicating the genes/environment distinction and the value of heritability analyses and the notion of a norm of reaction, which are built on the basis of such a distinction. He takes on the conviction of Lewontin and DST that genetic determinism is not as dead as it might seem and that someone still needs to make the stake-in-the-heart move (Oyama, 2000).

In a chapter with a rather flamboyant title (Kitcher, 2001) contributed, precisely, to the second of two volumes published in honor of Lewontin (Singh et al., 2001), Kitcher faces the challenge of what he is going to describe as “both versions of the transinteractionist^3^ approach” (p. 408). Starting with a detailed analysis of the notion of norm of reaction, he disputes both Lewontin’s and Oyama’s position and their criticism of the conventional interactionist interpretation. In his view, there is no fundamental error that explains the persistence of determinism in biology, and thus there is no need for a reconceptualization of parts of it, as Lewontin or DS theorists have suggested in different ways. Rather, he thinks, it persists because biologists misapply correct views.

He attributes biologists themselves a preference for simple over complex explanations and a certain difficulty in explaining complex science to the public. However, he admits that a serious consequence of this inability is precisely the widespread persistence of the idea of genetic determinism. He adds that, therefore, he understands Lewontin’s and Oyama’s motivation to call for a new approach. Nevertheless, he fears that the radicalism of their indictment and alternative view helps to strengthen genetic determinism rather than weaken it. He is also afraid that it distances scientists from working efforts to explore non-genetic factors.

Kitcher examines and opposes four main claims defended by “trans-interactionists” (where he places Lewontin, Oyama, Griffiths, Gray) against genetic determinism, three of which are particularly relevant from the point of view of parity: (i) that organisms and environments are inter-dependent, (ii) that there is developmental noise in the production of phenotypes and this undermines the partitioning of causes in norms of reaction, (iii) that the singling out of genes against background environmental conditions is a misguided abstraction from a complex causal situation, and (iv) that the notion of a “gene for a trait” cannot be coherently reconstructed. The third claim is very relevant here. Since interactionists acknowledge that many factors intervene in development, they support a principle of *causal democracy*:

**Causal democracy:** if the effect *E* is the product of factors in set *S*, then, for any *C* ϵ *S*, it is legitimate to investigate the dependence of *E* on *C* when the other factors in *S* are allowed to vary.

This can be implemented by taking *E* to be a phenotypic trait, *C* to be a particular genotype, and *S* to be a large set of factors in the total environment. In this case, the principle renders legitimate the strategy that investigates the dependence of a trait on a genotype while allowing other factors to vary just as much as it renders legitimate the strategy of investigating the dependence of the trait on some environmental factor, allowing the genotype to vary. Technically, the principle allows for various ways of conducting causal analysis. But if this is the case, then the principle gives no special privilege to representations that put the role of genes at the forefront. But even more importantly, and for the same reasons, the principle does not render norms of reaction incongruent or illegitimate. In his view, “we can move on from the blanket charge that any kind of separation out of causal factors does violence to causal complexities of development” (Kitcher, 2001, p. 404).

On the other hand, he advocates a pragmatic and pluralistic stance that embraces a very rigorous and restrained use of every technique that biologists have at hand, both because they are helpful and valid to investigate, at least partially, well-formulated questions and specific and very concrete issues (staying away from easy and rapid conclusions) and because we do not have other means (pp. 407–408). Referring to hypothetical different set of models, he regrets that “neither Lewontin’s “dialectical biology” nor the “developmental systems theory” pioneered by Oyama offer anything that aspiring researchers can put to work” (p. 408).

Oyama has protested in a detailed rebuttal explaining why she thinks that Kitcher misrepresents both the positions of standard interactionism as well as those of its critics (Oyama, 2001, p. 179) but we will not follow this path. Instead, we will now bring here another response that confronts directly Kitcher’s skepticism about the prospects of critical approaches to offer alternative but feasible avenues of research. Following the trend for flamboyant titles, Griffiths (2006) supports Oyama’s view that there is an inherent theoretical problem in the way we conceptualize genes and, referring to her famous quote mentioned above, he says that “[P]roof that developmental information is not localized in the genes is the ‘stake in the heart’ that will lay the vampire of genetic determinism to rest” (Griffiths, 2006, p. 176). In order to argue for this claim, he examines “the fallacious ways of thinking about genetic causation that make up genetic determinism, considering that they are the natural consequence of attributing semantic properties to the gene” (pp. 177–189). Then, he adds data from an empirical survey of biologists that shows “an apparent association between endorsing informational representation of the gene and being relatively uninterested in contextual effects on gene expression” (p. 177, pp. 189–190). And, finally, he will try to prove Kitcher to be mistaken by disclosing what he considers is “a substantial research tradition in developmental psychobiology that fits the prescriptions of developmental systems theory” (p. 177, pp. 190–192).

So, what is at stake is, on the one hand, the clarification of the epistemological and sociopolitical implications of the concepts and theories that are embraced by each approach (avoiding potential misunderstandings) and, on the other, the assessment of the heuristic and practical possibilities for research that they, respectively, open up. The second response we bring to this work directly addresses both of them.

### An Empirical Defense of Interactionism

In a recent book, *Behaving. What’s Genetic, What’s Not, and Why Should We Care?* (2016), Schaffner (2016) selects Behavioral Genetics as the scientific research area serving as assessment target for what he calls the developmentalist challenge in “the discussion about genes and behaviors and the nature-nurture controversy” (p. 2). In this book Schaffner gathers together, and elaborates further, previous work he had been producing over the years and which has been published in scattered articles and book chapters. Importantly for our purposes in this paper, the main thesis of that book is that “… only by examining quite recent work at the interface of molecular genetics, neuroscience, and behavior can some of the controversies raised by the developmentalist challenge be clarified, and at least partially settled” (p. 74).

Addressing the two issues we have said were at stake here, Schaffner offers us, on the one hand, elements to clarify potential allegations of misunderstanding through a detailed analysis of the conceptual tenets that, according to the author, most (if not all) critics share regarding those previously mentioned discussion and controversy. Such a detailed examination is built stepwise through a careful characterization of 11 theses that allegedly separate what he calls *developmentalist* (this is, our “post-interactionist”) positions from traditional views (p. 2), covering 7 “sins” about causation and 4 “mistakes” about the nature-nurture relation^4^.

On the other hand, Schaffner offers a concrete way around the challenge we have seen in the previous section concerning the heuristics of the post-interactionist alternative by confronting it against scientific practice in behavioral genetics, specifically the work on the behavior of *C. elegans*, and psychiatric genetics. He does so through the definition of 8 rules that connect genes and behavior, which are derived from his survey of empirical work carried out over the years on that model organism: *Eight Rules relating Genes (through Neurons) to Behavior in C. elegans*. Those general principles that allegedly govern the relation between genes and behavior in *C. elegans* research are, according to Schaffner, the following (pp. 82–93): many genes → one neuron; (ii) many neurons → one type of behavior; (ii) one gene → many neurons (pleiotropy); (iv) one neuron → many behaviors (multifunctional neurons); (v) stochastic [embryogenetic] development → different neural connections; (vi) different environments/histories → different behaviors (learning/plasticity, short-term environmental influence); (vii) one gene → another gene …→ behavior (gene interactions, including epistasis and combinatorial effects); and (viii) environment → gene expression → behavior (long- term environmental influence). Schaffner indicates that the arrow (→) can be read as “affect(s), cause(s), or lead(s) to” (p. 93).

Once the conceptual positions in conflict are unambiguously specified and the general principles of a sensitive area of empirical research are detailed, Schaffner proceeds to assess how well do the classical and the developmentalist approaches succeed in this confrontation. In other words, the very test or challenge posed by Kitcher: “What do successful research programs in *C. elegans* area tell us about the soundness and applicability of these concepts?” (Schaffner, 2016, pp. 95–96).

In order to do that, Schaffner summarizes the 5 concepts that he thinks are central to developmentalist approaches in opposition and as response to the 11 theses that characterize traditional approaches (its seven deadly sins and four major mistakes). Those five core concepts found in the developmentalist challenge are: (i) *Causal parity*, in the sense that genes are on a par with other factors: (ii) *non-preformationism*, denying that traits are in any sense represented in the genes; (iii) *contextualism*, assuming that genes have no meaning outside of a context including other genes and encompassing circles of environment; (iv) *indivisibility*, in the sense that the effects of genes and environment cannot be distinguished in the traits, and (v) *unpredictability*, claiming that not even a total knowledge about genes and environment allows the prediction of traits (implying their emergent nature) (pp. 95–98).

The final result is quite interestingly a mixed one showing, on the one hand, that the extreme complexity of the relations could render traditional approaches to genetic explanation inadequate and, on the other hand, that some of the alternative general principles are not always and completely satisfied either, leaving ample space for more nuanced and intermediate research strategies and conceptual explanations. Although he questions the indivisibility and unpredictability theses, he accepts the causal parity thesis:

“No *C. elegans* investigator ever thinks genes act alone (…) Thus, *causally*, genes have parity with other molecules as severally necessary and jointly sufficient conditions (to produce traits), but *epistemically* and *heuristically*, genes do seem to have a primus inter pares status, even in an increasingly ‘epigenetic’ age” (p. 96).

## Discussion: Bringing the Philosophy of Causation to the Debate

Causal concerns run through the entire debate. As we have seen, early views, such as Oyama’s, are committed to causal parity. The dialectical view, in turn, has as one of its main tenets the idea that the amount of variation in a phenotype cannot be partitioned into different and separable causes. On the other hand, we have seen that Kitcher’s criticisms against post-interactionists are partially based on his claim that, if genetic determinism persists, it is not so much due to actual views on genetic causation, which are not flawed, but to simplifications of such views. Schaffner’s examination of the post-interactionist thread is also centered around causation, as he associates the thread with the “sins” about causation that we have reviewed. As it is known, post-interactionists endorse a thesis of causal parity; however, it is interesting to note that critics of this tradition such as Schaffner and Kitcher do not straightforwardly reject (some version of) it. Indeed, Kitcher accepts the principle of “causal democracy,” although for him this principle says less about the biological phenomena than about the investigative strategies that scientists are justified in following. And Schaffner also concedes that, in *C. elegans* research, *causal* parity is admitted in principle –even if genes are often privileged *epistemically* and *heuristically*.

Thus, the debate around interactionism leads us to discuss issues in the philosophy of causation. One would expect the fuss to originate from the lack of consensus with regard to how to deal philosophically with complex causal relations in biology –specifically, in what respects the joint occurrence of genetic and environmental factors. Unfortunately, the views on causation upon which claims about interactionism are grounded are not made explicit in this debate. In this section, we contend that much of the debate around interactionism is based on the drawing of the wrong conclusion from causal complexity, that is, **that causal complexity excludes causal analysis**.

As can be seen in the overview provided in previous sections, it seems that many philosophers of biology are too puzzled and pessimistic about polygeny in development. From this, many of them draw what we think is an unwarranted conclusion: that causal complexity *excludes* the possibility of characterizing, distinguishing, or comparing among causal contributions. Interestingly, this seems to be an issue common to interactionism and post-interactionism alike: the two views fail to go beyond very simple statements about single causes not being sufficient to produce an effect, but readily subscribe to a thesis of causal parity.

This conclusion, however, should be deemed highly pessimistic: it condemns the entire philosophical project of performing causal analysis of natural phenomena to failure, to the extent that causal complexity is more of a rule than an exception in the natural world. But, has anyone in the interactionist debate provided solid reasons to endorse such a pessimistic conclusion? We claim that they have not and, in the next three sub-sections, we will try to argue why.

First, turning to Keller’s (2010) conceptual and historical reconstruction, we will highlight the apparently (but not truly) obvious relevance of clarifying epistemologically what is originally at stake in the debate. Then we will expand the scope in order to insert the postulates of the debate within the more general issue of causal selection, where such an epistemic option finds its rationale. Finally, we will explore the prospects a more ontologically engaged approach could offer.

If our arguments in this section hold, in the last one, we will conclude that a great deal of the difficulty involved in the debate about interactionism stems from the wrong inference we have identified, and we will extract some consequences for the project about inter-identities (CfP, 2019).

### Clarifying the Original Confusion at the Source of the Debate

Let us return for a moment to Keller’s remark, mentioned in the introduction, on the perdurability of the issue(s) at stake here. Despite the discussions we have briefly depicted using some representative positions and, allegedly, the resulting clarification of the questions involved, Keller finds nevertheless, and to her amazement, that the debate on interaction remains practically the same (particularly in the wider public realm). This, for the most part, simply restates the limitations of the standard interactionist solution, as many critics have already reminded us.

In short, the point is that any easily manageable notion of interaction requires pre-existing causal factors that are theoretically separable and this is precisely what we do not get in the complex and intricate processes leading to the development of biological and behavioral traits (Keller, 2010, p. 6). This should be just a logical point beyond debate: causes that are not mutually independent and interact in such entangled ways “simply cannot be parsed” (Keller, 2010, p. 7^5^). But in fact, it is not:

*“If all that was at issue in the nature-nurture debate was a comparison of the contributions of nature and nurture to individual development (…) [critics] are of course correct: this question is meaningless (‘we can’t readily separate one from the other’), and the debate could indeed be said to be over. But unfortunately, the question of what the nature-nurture debate is about is not so easily settled”* (Keller, 2010, pp. 8–9).

And, accordingly, the fate of interactionism as such is not either. The reason for this is that not all the people are talking all the time about the same, partcularly when we turn to scientific practice. For instance, “… to population geneticists, the debate is **not about** relative contributions to **individual traits**, **but about** contributions to the **variation within a population**. Still others think of it as being **about** the relative importance of the contributions of nature and nurture to **differences between individuals**” (p. 9, bold added).

This is why she states from the very beginning that the problem lying at the core of the issue is that the very question involved, “what is the nature/nurture debate about?” (p. 1), is not a clear one but, instead, implies a variety of entangled questions which are ambiguously posed. She adds that the very language of genetics is particularly responsible for that situation. She elaborates on these two qualifications in the meaning of the debate, introduced in her quote above (moving “from trait to trait difference” and “from individuals to populations”), through a detailed historical review of the introduction and transformation of the concepts of genetics. Since it helps us to establish the basis of our argument we will briefly summarize the main points of this historical survey.

First, we find the claim that seeing nature and nurture, genetics and environment, as separate causal factors has a historical origin. Keller traces back this separation, which might be at the root of the problem, to the end of the nineteenth century, already with Darwin and Spencer but more clearly then with Galton.

Keller locates at this moment the turn in the usage of the phrase “nature and nurture,” which she has briefly traced from Shakespeare to J. S. Mill and that, according to her (and other scholars with whom she agrees), did not imply the sense of opposition and separation that would acquire from then on. She claims that it was only after Galton that the new connotation became clearly established and that it was already entailed by the re-signification of the notion of heredity that Darwin and others are engendering. The turn involves three elements. One is the aforesaid change in the notion of conceptions of heredity that brought about a new alignment between innate (or inborn) and hereditary. Another is the subsequent internalization and substantiation of heredity. And the third is the introduction by Galton of a particulate theory on inheritance that will accomplish the definition and instantiation of the separation (Keller, 2010, pp. 20–27).

Having established this starting point, she then goes on to explain the reformulation already made by Fisher at the beginning of the twentieth century that will reveal, as early as then, the source of the current clash or entanglement of meanings. The tension would be that between Galton’s approach to the new distinction and the more technical application of it in population genetics. She thinks that clarifying how those terms and concepts have been used and are used in (population and developmental) genetics could help both to understand the problem (why it is so resistant) and to reformulate when and how the question might be meaningful.

“*… Galton’s hope of sorting genetic from environmental influences would need to be recast in two important ways. First, it was necessary to reformulate the question of causation in terms of trait differences rather than in terms of traits *per se*, and second, it was necessary to turn form the analysis of heredity in individual lineages to the analysis of heredity in populations. Only if we ask a statistical question about the relative contributions of variations in genetics and in environment to our differences–rather than their relative contributions to the process that make us what we are–would we have a question that makes sense, and furthermore, one that we might be able to answer”* (Keller, 2010, pp. 31–32).

The consequence of these distinctions is the possibility of understanding why even if we are not able to parse the causal contributions of genetics and environment to individual traits we might still be able, in some cases, to statistically parse the causal contributions of differences in genetics and environment to differences in traits averaged over a population (Keller, 2010, pp. 12).

Nevertheless, she will review and bring up examples to show how difficult it turns out to be, even for specialists, to keep up these distinctions sharp, both in technical or popular science discourse. This would, at least in part, explain the “unreasonable persistence” of the debate: the root of the problem is inserted in the very language of particulate genetics^6^. Even if the science has changed, that is, genetics has become much more complex, she claims that language has lagged behind, allowing those slippages of meaning. This is a judgment, incidentally, that we could extrapolate to other areas of research.

The interesting point for us here to reinforce the idea that we have been developing in the previous sections: beyond, or due to, the inseparability of factors implied by most of the explanations resorting to the concept of interaction in biological, behavioral and cognitive sciences, it is necessary to specify if and when distinctions between causal factors can be made under certain conditions (so rendering interaction an effective epistemic resource).

In order to achieve such disciplinary generality (i.e., to fulfill the goal of our paper which is to offer some informed forewarning to the attempts “to understand and model complexity in the living, the cognitive and the social domains” in terms of interaction) this requirement cannot be justified by merely looking at a particular discussion in biology and the details of its historical conceptual development (however decisive they are); instead, a broader (causal) philosophical approach is required.

We should first take a quick look to the problem of causal selection that allows us to move to this broader approach. Such a frame helps us typify the option resulting from Keller’s analysis as an example of how to make distinctions among causal factors attending to epistemic needs or pragmatic choices. Once we establish this, we can bring up the much more difficult and pressing issue of whether we may attempt (and to what extent) to go beyond and ground ontologically those differences among causal factors.

### The Problem of Causal Selection

As mentioned before, causal complexity is, to a great extent, due to the fact that many (if not most) effects of interest are brought about by a multiplicity of causal factors (i.e., are multi-causal or polygenic). This is counteracted by the fact that, even when we find that multiple factors are relevant for the occurrence of a given phenomenon, we tend to treat a subset of those as *the cause* or *the causes* of the phenomenon. The rest, we regard as mere *background conditions*. Causal selection is this practice of selecting certain factors over others. To use a classical example, consider the lighting of a match. We accept that several factors are relevant to the lighting of the match: the fact that Mary stroke it, the presence of oxygen in the room, the dryness of the match, and so on. Yet, in response to the question “What caused the match to light,” we tend to pick Mary’s striking of the match as the cause. Presence of oxygen and dryness are regarded as background conditions. Such a selection *practice* is pervasive, both in the sciences and in everyday life. In both everyday experience and scientific investigation, we simply do not cite every possible factor as causes of a phenomenon of interest, but instead select some of them. Yet, as we said, both oxygen and the striking of the match are relevant for the effect. Thus, the *problem* of causal selection minds the grounds for discriminating between genuine causes and mere background conditions, what is the nature of these grounds, or whether the practice of causal selection is irreducibly ungrounded (Broadbent, 2008; Franklin-Hall, 2015; Ross, 2018; Baxter, 2019).

In this context, a view that is considered the consensus in philosophy (e.g., Schaffer, 2016) is the one advocated by Mill (1974 [1843]). Milleanism is the view according to which there are no ontological differences between causes, and these only differ ontologically from non-causes (Mill, 1974 [1843])^7^. Any distinction between what we call a cause, and what we call a background condition, responds exclusively to the interests of the investigation (or to the interests of everyday causal judgments).

This view has more recently been contested. Indeed, many have argued against Milleanism on the grounds that important *objective* distinctions can and need to be drawn among causal relations. Recent contributions claim that causal selection is too consistent and systematic to be a merely pragmatic affair (e.g., Hart and Honoré, 1985; Ross, 2018), and that the mere fact of the multiplicity of causes does not imply that they are ontologically indistinguishable (e.g., Waters, 2007). Numerous attempts have been made to grasp the distinction between causes and background conditions such as those involving necessity/sufficiency considerations, contrasting and counterfactual considerations, a notion of causal control, and/or combinations thereof (Ducasse, 1926; Mackie, 1965; Broadbent, 2008; Ross, 2018, forthcoming).

In fact, this problem of causal selection constitutes the angle from which some philosophers have argued —if not against post-interactionism all together—against the causal parity thesis. In the next section, we discuss such an argument and use it to make our case that, as stated previously, much of the debate around interactionism stems from drawing the wrong conclusion from causal complexity, namely, that it resists the characterization, comparison, and distinction of causal relations.

### Interactionism, Post-interactionism, and Causation

It is acknowledged, in the recent philosophy of causation, that we often compare causes within certain domains and apportion varying degrees of causal responsibility. The investigation of such practices is an important philosophical project for clear reasons. Often, one is not exclusively concerned with *detecting* causal relations, but with the *characteristic features* of particular causal contribution. The analysis of the respects in which causal relations differ can be made on the basis of several properties, for example, strength or degree of the causal contribution, proportionality in the grain of description for a causal structure, or stability of the relation over a range of different background circumstances (see Northcott, 2005; Braham and van Hees, 2009; Woodward, 2010).

This is indeed the point of view of a few philosophers who opted to tackle questions of interactionism and the nature/nurture debate from a strict causal angle, in particular, through a discussion of the Causal Parity Thesis (CPT), i.e., the uncompromising upshot of the principle of causal democracy introduced above (section “The Heuristic Value of Post-interactionism”). It has been noted that CPT expresses a particular stance with regard to the problem of causal selection, namely an extreme Millean exclusion of any relevant causal distinction. Critics of causal parity argue on the contrary that objective distinctions can be drawn which would ground the view that factors are *not* causally on a par (Weber, 2005, forthcoming; Waters, 2007; Woodward, 2010). Let us see briefly, as a clarifying illustration, how the attribution of causal specificity in the case of DNA is argued for within the interventionist approach to causation.

In particular, critics have invoked the concept of causal specificity to make the case that DNA is *ontologically different*. This concept has been spelled out in a few related ways. For Waters (2007), it has to do with the possibility that many different changes in the cause led to many different changes in the effect. Woodward (2010) presents it as a matter of the grain of influence or control that (idealized) interventions on a cause enable over an effect. This is illustrated with a radio analogy that compares the *on/off switch* to the *tuning dial*: there are many possible positions for the dial, many possible radio stations, and a relationship holding between both that enables a fine-grained control over what is heard on the radio. I can intervene on the dial in many ways so as to tune various different stations. By contrast, the on/off switch, while causally relevant to whether *a* station is received, has little influence on *which* one is received. One cannot intervene on the on/off button in order to tune the different stations, but only to turn the radio either on or off. When causal relations have this property, they can be exploited in various ways, allowing a fine-grained control of what happens to the effect. Claims that DNA is a highly specific cause (or a dial-like cause) of protein synthesis mean that: “there are many possible states of the DNA sequence and many (although not all) variations in this sequence are systematically associated with different possible corresponding states of the linear sequences of the mRNA molecules (…). Thus, varying the DNA sequence provides for a kind of fine-grained and specific control over which RNA molecules or proteins are synthesized” (p. 306). Claims that the polymerase is not specific mean that interventions on it do not provide fine-grained control. The role of the RNA polymerase, by contrast, is switch-like.

The specificity argument against parity states that the customary singling out of genetic factors in a large range of causal explanations in biology does not simply follow from the needs and interests of particular investigations, but it (in addition) reflects some objective aspect of the world. Whilst the reasons why we happen to be interested in inquiring about the cause(s) of some effect rather than another might well respond to all kinds of pragmatic and subjective reasons, the same is not true for the investigation of the causes. Once an effect has been specified, the question about its causes is an ontological one, “fixed by ontology” (Waters, 2007). Waters argues that, similarly, once the causes of a given effect have been identified, their characteristics (e.g., whether they bear a specific relation to the effect) are, too, ontological ones.

We need to take stock from this discussion. The problem with the literature on interactionism is that, while it revolves around very general claims about causation, it is rarely made explicit, if ever, which is the working causal-philosophical viewpoint. In particular, it tends to ignore the problem of causal selection and polygeny or is presented as an insurmountable issue that resists any possible analysis. This contrasts with alternative developments and points of view in philosophy of causation. In our view, the debate will not move forward unless the philosophy of causation is properly taken into account, be it to endorse explicitly epistemic and pragmatic solutions or more demanding ones, such as those that entail an ontological commitment.

## Conclusion and Prospects for the Project

In the first part of this paper, we have examined the ongoing debate on interactionism in the philosophy of biology. More specifically, we have first presented the justification behind the move toward an interactionist consensus. We have then introduced a selection of the main concerns raised by post-interactionists against the traditional interactionism that, in their view, demand an alternative, perhaps stronger approach. Next, we have also considered some representative views opposing the claim that a different approach is needed and, in this sense, vindicate a more standard form of interactionism.

The appraisal of the diverse positions on interactionism brings us to the point where we cannot ignore the warnings of its critics but, at the same time, we need to be able to face also a Kitcher-like minimalist kind of defense:

*“… for the present, the interactionist’s claim is simply that we should not suppose that efforts to investigate the effects of some factors, while others are allowed to vary, are incoherent or illegitimate. Complex causal situations do not demand that we perform the impossible feat of considering everything at once; rather they challenge us to find ways of making these factors manageable”* (Kitcher, 2001, p. 404).

In the second part of this paper, we have brought this sort of defense of interactionism under the light of discussions in the philosophy of causation that, in our view, should be directly tackled by those involved in the interactionism debate or those willing to adopt an interactionist strategy in their research.

Even if the philosophical debate around interactionism as such is still worthy of further discussion, and things are far from settled, our analysis in this paper already allows us to extract some valuable conclusions that could inform the various efforts to develop models of “complex systems that comprise “inter-identities” (CfP, 2019).

One first conclusion is that, when exploring “issues of identity in biological, cognitive and social, biomedical, educational and political systems” (CfP, 2019), we should not ignore the implications of the nature/nurture controversy and its accompanying debate on interactionism that has arisen in evolutionary and developmental genetics (and elaborated and pursued within the philosophy of biology), which we have surveyed here.

A second one is that, in those areas of research, aside from addressing the more substantial or empirical issues, an explicit methodological and heuristic task has to be undertaken in each instance and for every explanation or research purpose: to identify and justify whether and how diverse causal factors are going to be distinguished, whether and under what conditions such causal factors are going to be parsed and how their mutual relation is going to be accounted for. This is so because, to remain within the limits of the so-called interactionism consensus, as a default and non-committed hollow stance, entails the endorsement a Millean view on causality and, then, this heuristic or pragmatic challenge becomes even more decisive. Setting the conditions of possibility to obtain the epistemic benefits granted by a heuristic stance (see Ferreira Ruiz and Umerez, 2018) becomes, therefore, the minimum requirement to be met by any explanatory and research endeavor forced to incorporate complex causal contributions that interact.

That said, a third conclusion is that we need not restrict the discussion around interactionism and the nature-nurture distinction to epistemic or pragmatic issues. Rather, this debate can benefit from other philosophical projects, especially, in the metaphysics of causation. In particular, we should not overlook the issue on causal selection that we have shown to beset the interactionist debate whenever multiple and complex causality is involved. Accordingly, it would be advisable to disclose and make explicit, as clearly and exhaustively as possible, any causal selection decision and the underlying grounds for this (be it purpose-specific or general, instrumental or ontologically committed, conditional or unconditional). Similarly, when addressing those debates in the philosophy of biology, we must seriously consider the possibility of drawing various kinds of distinctions among causal relations.

Finally, it must be noted that while it will still be the predicament of every researcher to explore and determine how far they can go in order to characterize an identity that is developed in interaction, as a preliminary step, it will be necessary to revise the assumptions around interaction and interactionism–as we have attempted to do here. It will soon prove extremely difficult to move forward in characterizing identity in interaction if the interaction and joint contribution of multiple causal factors is simply assumed to be an insurmountable obstacle to causal analysis.

## Author Contributions

All authors listed have made a substantial, direct and intellectual contribution to the work, and approved it for publication.

## Conflict of Interest

The authors declare that the research was conducted in the absence of any commercial or financial relationships that could be construed as a potential conflict of interest.

## Footnotes

1 We should make clear from the beginning that, when dealing with interactionist views in the philosophy of biology (but not exclusively), the debate is not about the causal roles of *parts* in (dynamic) systems and the ways to identify and distribute their relative contributions to the performance of the whole system, but one about the aggregated causal roles of the very systems themselves, which are allegedly separated and, often, qualitatively different, in the production of an outcome (such as genes and environment generating a particular phenotypic trait, in the canonical case). We thank a reviewer for pointing out the importance of this clarification.

2 This quote will gradually become the source of frequently repeated catchphrases and several striking paper titles.

3 Kitcher uses the expression “transinteractionism” for what we call “post-interactionism.”

4 He first details the *Seven Deadly Sins of Causation*, which are:

(i) the acceptance of the nature-vs-nurture dichotomy,
(ii) the principle that one gene causes one behavior,
(iii) the description of genes as blueprints,
(iv) the assertion that DNA sequences contain the essence of behavioral information,
(v) the belief that genes cause behavioral traits fairly directly,
(vi) the idea that genes are the root cause of behavior, and
(vii) the view that a gene produces a single, clear, and specific phenotype (pp. 71–72).

These *sins* are coupled to *Four Major Mistakes of Classical Approaches to Nature and Nurture*, including:

(i) the conviction that behaviors divide neatly into innate and learned classes,
(ii) the confidence that empirical studies can disentangle the effects of heredity into specific percentages,
(iii) the consideration of analysis of variance (ANOVA) methods as powerful tools that reveal developmental effects, and
(iv) the idea that a “heritability” is an excellent summary statistics (pp. 72–73).

The description of each one of these disputed theses is followed by the formulation of the direct alternative or contrary view by the developmentalist approaches.

5 See also Lewontin (1974) as introduced and quoted above (section “Lewontin’s *Dialectical Biology*”).

6 There is another very important point that may also in part explain such persistence but we are not going to be able to pursue it here: “… the fact that (…) the debate does seem to capture a number of issues that many people want to know about” (Keller, 2010, p. 73). This opens up a very interesting analysis about the concept of phenotypic plasticity and the possibilities of rethinking and expanding the notions of inheritance and heritability, together with the social and political issues such an undertaking raises (Keller, 2010, pp. 73–84).

7 “Nothing can better show the absence of any scientific ground for the distinction between the cause of a phenomenon and its conditions, than the capricious manner in which we select from among the conditions that which we choose to denominate the cause. However numerous the conditions may be, there is hardly any of them which may not, according to the purpose of our immediate discourse, obtain that nominal pre-eminence” (Mill, 1974 [1843], p. 329).
